# Prognostic role of the platelet to lymphocyte ratio (PLR) in the clinical outcomes of patients with advanced lung cancer receiving immunotherapy: A systematic review and meta-analysis

**DOI:** 10.3389/fonc.2022.962173

**Published:** 2022-08-22

**Authors:** Ke Zhou, Jie Cao, Huahang Lin, Linchuan Liang, Zhongzhong Shen, Lei Wang, Zhiyu Peng, Jiandong Mei

**Affiliations:** ^1^ Department of Thoracic Surgery, West China Hospital, Sichuan University, Chengdu, China; ^2^ Western China Collaborative Innovation Center for Early Diagnosis and Multidisciplinary Therapy of Lung Cancer, Sichuan University, Chengdu, China

**Keywords:** platelet to lymphocyte ratio, advanced lung cancer, immune checkpoint inhibitor, prognosis, immunotherapy, biomarker, meta-analysis

## Abstract

**Background:**

It remains controversial whether the platelet to lymphocyte ratio (PLR) serves as a potential indicator for the efficacy of immunotherapy in advanced lung cancer. This meta-analysis aimed to address this concern.

**Methods:**

Up to March 2022, we searched PubMed, Embase, Web of Science and the Cochrane Library to retrieve potentially eligible articles. Combined hazard ratios (HRs) and 95% confidence intervals (CIs) were estimated to assess the relationship between PLR and progression-free survival (PFS) as well as overall survival (OS), while the combined odds ratios (ORs) and 95% CIs were estimated to evaluate the relationship between PLR and the objective response rate (ORR) as well as the disease control rate (DCR). Subgroup analyses were further performed to detect the source of heterogeneity and potential predictive value of PLR in different groups in terms of OS and PFS.

**Results:**

A total of 21 included studies involving 2312 patients with advanced lung cancer receiving immunotherapy were included. The combined results suggested that elevated PLR was associated with poorer OS (HR=2.24; 95% CI: 1.87-2.68; I² =44%; P=0.01) and PFS (HR=1.66; 95% CI: 1.36-2.04; I² =64%; P<0.01). Furthermore, elevated PLR showed a lower ORR (OR= 0.61; 95% CI: 0.43-0.87, I²=20%; P=0.29) and DCR (OR= 0.44; 95% CI: 0.27-0.72, I²=61%; P=0.02). In subgroup analyses, pretreatment PLR was significantly associated with adverse OS and PFS. The same results were observed in different PLRs in terms of cutoff value (>200 vs. ≤200). Furthermore, high PLR was significantly associated with poor OS and PFS in advanced non-small cell lung cancer (NSCLC); however, PLR was not associated with OS and PFS in advanced small cell lung cancer (SCLC). In addition, PLR predicted poor OS irrespective of regions and types of immune checkpoint inhibitors (ICIs).

**Conclusion:**

On the whole, patients with low PLR had better OS and PFS, as well as higher ORR and DCR when receiving immunotherapy in advanced lung cancer especially for advanced NSCLC. And further investigations are warranted to confirm the prognostic value of PLR in advanced SCLC.

**Systematic Review Registration:**

https://www.crd.york.ac.uk/PROSPERO/, identifier CRD42022315976.

## Introduction

Lung cancer, as one of the most common malignant tumors in the world, ranks first in both morbidity and mortality (1). Surgical resection remains the optimal choice for early-stage lung cancer (2). Unfortunately, most lung cancer patients are identified in advanced stages, and postoperative adjuvant therapies become necessary for these patients (3). To date, postoperative adjuvant therapies have enriched with targeted therapy and immunotherapy in addition to traditional chemotherapy and radiotherapy. In particular, immune checkpoint inhibitors (ICIs), such as nivolumab, have yielded encouraging results in advanced lung cancer by showing superior clinical benefits even after the failure of other treatments (4). However, individuals show a different response to ICIs, and not all advanced patients are able to benefit from ICIs (5). Multiple biomarkers found before, including tumor mutational burden (TMB) (6), neoantigens (7) and programmed cell death 1 ligand 1 (PD-L1) expression (8), have shown great potential predictive values for immunotherapy. However, some reports demonstrated that patients with negative PD-L1 expression or low TMB could benefit from ICIs (9). Furthermore, it is complicated to acquire these data with an additional economic cost for patients. Therefore, it is urgent to find inexpensive and practical biomarkers associated with immunotherapy outcomes.

The systemic inflammatory response in cancer patients exerts an important effect on the development of tumors *via* certain inflammatory factors (10). PLR is known as the ratio of platelets to lymphocytes, and elevated PLR was proven to be associated with poor survival in many cancer patients (11, 12). In recent years, several studies have explored the relationships between PLR and immunotherapy in advanced lung cancer. Some of them claimed that PLR could predict the outcome of immunotherapy (13), while others reported negative findings (14). The role of PLR in predicting the efficacy of immunotherapy in advanced lung cancer remains controversial. To address this concern, we collected relevant publications and conducted a meta-analysis to assess the correlation between PLR and the efficacy of immunotherapy in advanced lung cancer.

## Materials and methods

We strictly followed the PRISMA guidelines (15) to conduct this systematic review and meta-analysis and registered it on PROSPERO (registration number: CRD42022315976). The PRISMA checklist was provided in **Supplementary Table S1**.

### Search strategy

Up to March 2022, we searched the PubMed, Cochrane Library, Web of Science and Embase databases to collect relevant articles evaluating the relationships between PLR and clinical outcomes of immunotherapy in advanced lung cancer. The search keywords were as follows: lung cancer, platelet lymphocyte ratio, immunotherapy, and immune checkpoint inhibitors. We provided a detailed online search strategy (**Supplementary Table S2**). Other eligible studies were retrieved from the references cited in the selected articles and relevant literature.

### Eligibility criteria

The inclusion criteria were as follows (1): advanced lung cancer including NSCLC or SCLC with recurrent, metastatic, unresectable situation or advanced stage (III or IV) (2); receiving any types of ICIs (3); reporting the value of the PLR before or after immunotherapy (4); study outcomes including overall survival (OS), progression-free survival (PFS), objective response rate (ORR) or disease control rate (DCR); and (5) the effect estimates and corresponding 95% CI were reported or could be estimated. The exclusion criteria were as follows (1): review, letter, meeting, abstract, full text unavailable; and (2) overlapping publications and repeated facilities.

### Data extraction and quality assessment

According to PRISMA statements, we extracted the title, first author, study design, year of publication, sample size, sex, region, median follow-up time, histology, PLR cutoff value, time to record PLR, types of ICIs, presence of driver gene mutations, including epidermal growth factor receptor (EGFR) or anaplastic lymphoma kinase (ALK), number of patients with PD-L1+ (tumor proportion score [TPS]>1), HRs for OS and PFS and rate for ORR and DCR from each included study. When multivariate analysis and univariate analysis results both occurred in one study, we chose the multivariate analysis results since they accounted for confounding factors and were more accurate. The Newcastle–Ottawa Scale (NOS) (16) was used to evaluate the quality of the included studies. NOS evaluated the quality of articles from three main aspects: selection, comparability, and outcome. High-quality studies were defined to attain at least six NOS scores. Two investigators independently extracted data and conducted NOS evaluations. If there was any disagreement about the results, a third reviewer was consulted to resolve the concerns.

### Statistical analysis

The endpoints in our study were OS and PFS, while the secondary endpoints were ORR and DCR. The definitions of these endpoints were described in previous articles (9, 17). We pooled all HRs of OS and PFS *via* fixed effects or random effects models. If the HRs were not reported directly, HRs with 95% CIs for OS and PFS were indirectly estimated from the Kaplan–Meier curves using the method reported by Tierney (18). The synthesis of ORs for ORR and DCR was conducted by the Mantel–Haenszel method *via* fixed effects or random effects models. Subgroup analyses were further conducted to detect the impact of the region, sample size, cutoff value, time point, median follow-up time, stage, ICIs types, the presence of mutations and proportion of patients with PD-L1+. All combined HRs in subgroup analyses were performed *via* a random effects model. Cochrane’s Q statistic and I² statistic were used to estimate heterogeneity between studies. A P value <0.05 for the Q-test and I² <50% were considered to indicate low heterogeneity. Sensitivity analyses were conducted by excluding studies one by one from the meta-analysis. Publication bias was assessed by Egger’s and Begg’s tests, and trim and fill funnel plots were further conducted to estimate the HRs after eliminating potential publication bias. P<0.05 was defined to indicate significant publication bias. For all combined results, P<0.05 was considered statistically significant. All statistical analyses were conducted by R software (version 4.0.5).

## Results

### Search results

**Figure 1:**
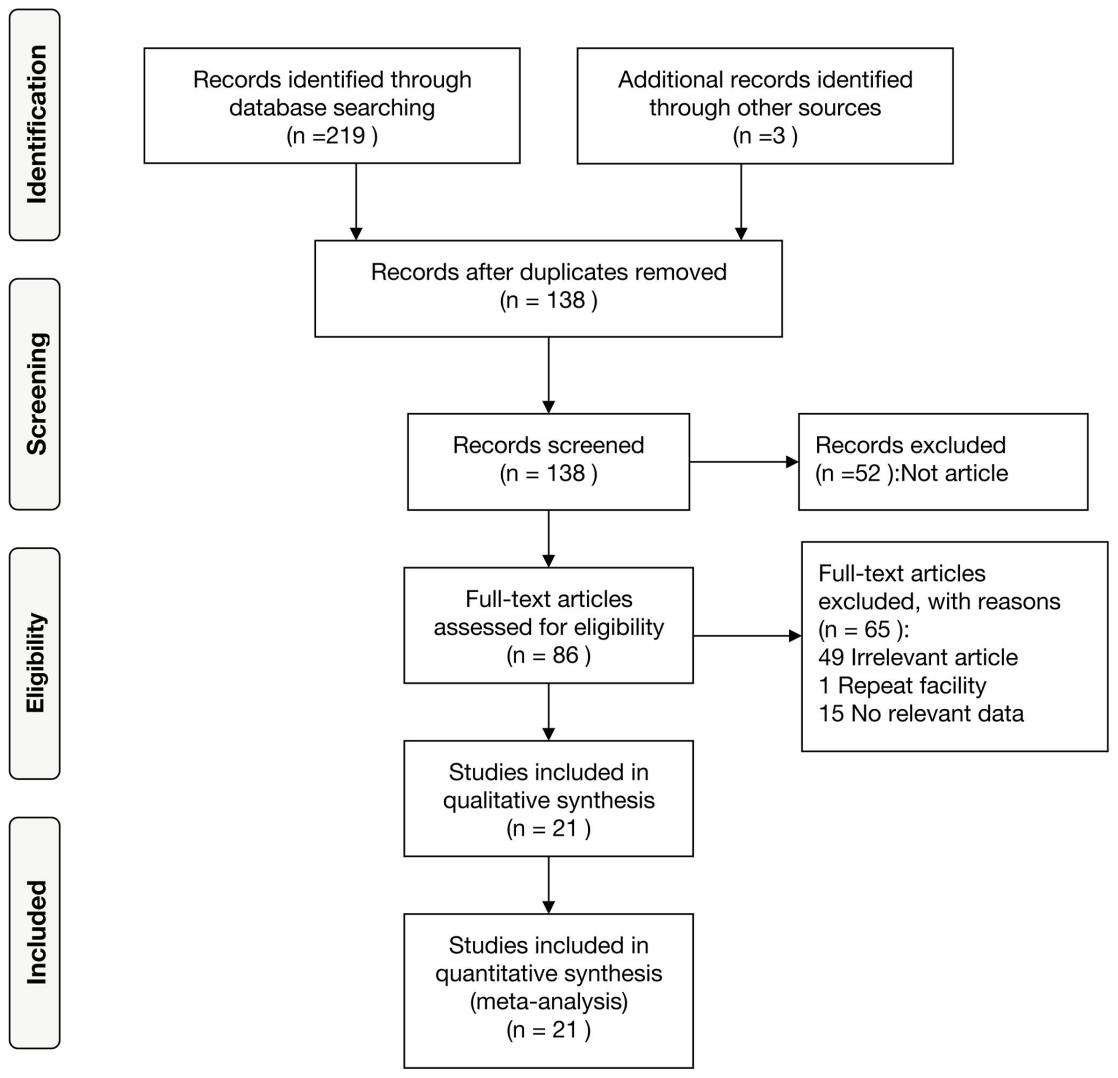
Flow diagram of the selection of the included studies.

After the initial search, a total of 222 studies were identified. Eighty-four of them were removed due to duplication, and 138 studies were left for subsequent selection. Ultimately, 116 studies were excluded, and the remaining 21 retrospective studies (13, 19–38) were included for further combined analysis. The detailed flow diagram of the included study process is shown in **Figure 1**. There were 21 studies involving 2312 advanced lung cancer patients treated with immunotherapy published from 2017 to 2022. The cutoff value of PLR ranged from 119.2 to 441.8, and the cutoff values of 15 articles were within 200. The baseline characteristics of the included studies were listed in **Table 1**. The detailed NOS values of the included studies were listed in **Supplementary Table S3**.

**Table 1:** Baseline characteristics of the included retrospective studies.

Study	Year	Region	Study type	Sex(M:F)	Sample size	Smoking(%)	Stage	SCC%	PDL1+(%)*	Mutation+^#^	Time to record PLR	Cutoff value	>cutoff	Type of ICIs	Outcomes	MFT	NOS value
Diem	2017	Europe	Retrospective	29:23	52	92%	aNSCLC	35%	46%	NM	Pre	262	NM	N	OS、PFS	NM	7
Svaton	2018	Europe	Retrospective	71:49	120	82%	aNSCLC	33%	NM	NM	Pre	169.1	71	N	OS	NM	7
Suh	2018	Asia	Retrospective	42:12	54	72%	aNSCLC	31%	20%	7	Post	169	16	N/P	OS、PFS、DCR	26.2	9
Liu	2019	Asia	Retrospective	33:11	44	66%	aNSCLC	30%	NM	8	Pre	144	26	N	OS、PFS	6.9	8
Pavan	2019	Europe	Retrospective	125:59	184	87%	aNSCLC	32%	45%	25	Pre	180	76	N/P/A	OS、PFS、AE	6	9
Dusselier	2019	Europe	Retrospective	44:15	59	NM	aNSCLC	20%	24%	NM	Pre/Post	262	8	N	OS	NM	7
Jiang	2020	Asia	Retrospective	66:10	76	79%	aNSCLC	45%	55%	NM	Pre/Post	168.13	26	N/D	OS、PFS、DCR	7.1	9
Katayama	2020	Asia	Retrospective	44:37	81	79%	aNSCLC	21%	46%	14	Pre	262	NM	A	OS、PFS	NM	6
Matsubara	2020	Asia	Retrospective	17:7	24	71%	aNSCLC	17%	38%	5	Pre	150	18	A	OS、DCR	NM	5
Petrova	2020	Europe	Retrospective	74:45	119	NM	aNSCLC	43%	100%	0	Pre	200	59	P	OS、PFS、DCR	NM	7
Russo	2020	Europe	Retrospective	137:50	187	87%	aNSCLC	46%	8%	5	Pre	200	54	N	OS、PFS、ORR、DCR	NM	7
Takada	2020	Asia	Retrospective	184:42	226	84%	aNSCLC	27%	51%	197	Pre	245	85	N/P	OS、PFS、ORR、DCR	13.7	9
Ksienski	2021	American	Retrospective	99:121	220	91%	aNSCLC	20%	100%	3	Pre	441.8	50	P	OS、AE	9.2	8
Park	2021	Asia	Retrospective	62:21	83	80%	aNSCLC	29%	100%	27	Pre	210	NM	P/A	OS	7.3	7
Pu	2021	Asia	Retrospective	134:50	184	67%	aNSCLC	37%	71%	NM	Pre	200	85	N/P	OS、PFS、ORR、DCR	9.2	7
Qi	2021	American	Retrospective	34:19	53	34%	aSCLC	NM	NM	NM	Pre	119.2	NM	A	OS	17.1	9
Gastaldo	2021	Europe	Retrospective	37:14	51	55%	aNSCLC	37%	100%	0	Pre	198	24	P	OS、PFS	6.93	9
Seban	2021	Europe	Retrospective	31:20	51	98%	aNSCLC	24%	100%	0	Pre	150	NM	P	OS、PFS	26.5	9
Xiong	2021	Asia	Retrospective	36:5	41	85%	aSCLC	NM	NM	NM	Pre/Post	169	21	N/P/A/T	PFS	NM	7
Holtzman	2022	Asia	Retrospective	200:102	302	89%	aNSCLC	17%	100%	0	Pre	169	157	P	OS	28.6	7
Wu	2022	Asia	Retrospective	78:23	101	72%	aNSCLC	35%	43%	9	Pre	176	49	IO	OS、PFS、ORR、DCR	NM	7

*The proportion of patents with PD-L1+(TPS>1); TPS, tumor proportion score.

^#^The study provided the number of patients with lung cancer carrying driver gene mutations, including EGFR or ALK

M, male; F, female; ICIs, immune checkpoint inhibitors; N, nivolumab; P, pembrolizumab; A, atezolizumab; T, toripalimab; IO, PD1/PD-L1 inhibitors; D, durvalumab; aNSCLC, advanced non-small cell lung cancer; aSCLC, advanced small lung cancer; SCC, squamous cell carcinoma; Pre, pretreatment PLR; Post, posttreatment PLR; PFS, progression-free survival; OS, overall survival; ORR, objective response rate; DCR, disease control rate; MFT, median follow-up time; NM, not mentioned.

### Relationship between the PLR and ORR/DCR

**Figure 2:**
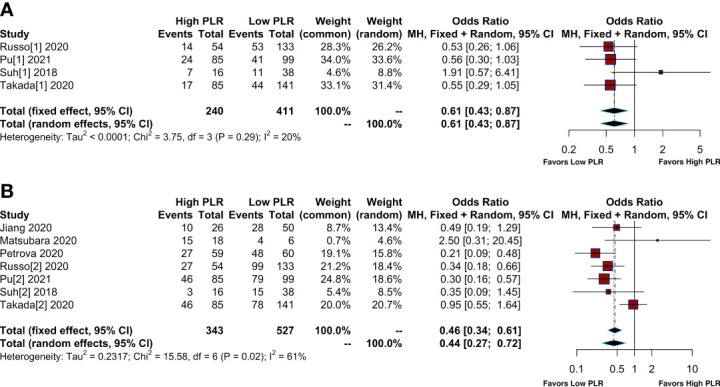
Meta-analysis of the relationship between different comparative models of the platelet to lymphocyte ratio (PLR) and response. **(A)** Meta-analysis of the relationship between PLR and objective response rate (ORR); **(B)** Meta-analysis of the relationship between PLR and disease control rate (DCR).

Twelve articles reported the number of patients who achieved ORR and DCR after treatment with immunotherapy. In a total of 1332 patients, 238 patients were considered to achieve ORR and 628 to achieve DCR, with percentages of 17.86% and 47.14%, respectively. Furthermore, 62 patients (12.54%) achieved ORR in patients with high PLR, while it was 149 (21.18%) in patients with low PLR. In addition, 174 patients (35.22%) achieved DCR in patients with high PLR, while it was 351 (49.74%) in patients with low PLR (**Supplementary Table S4**). The pooled OR for ORR and DCR were shown in **Figure 2**. Compared to patients with low PLR, patients with high PLR showed a lower ORR (OR= 0.61; 95% CI: 0.43-0.87, I²=20%; P=0.29) (**Figure 2A**) and lower DCR (OR= 0.44; 95% CI: 0.27-0.72, I²=61%; P=0.02) (**Figure 2B**).

### Relationship between the PLR and OS/PFS

**Figure 3:**
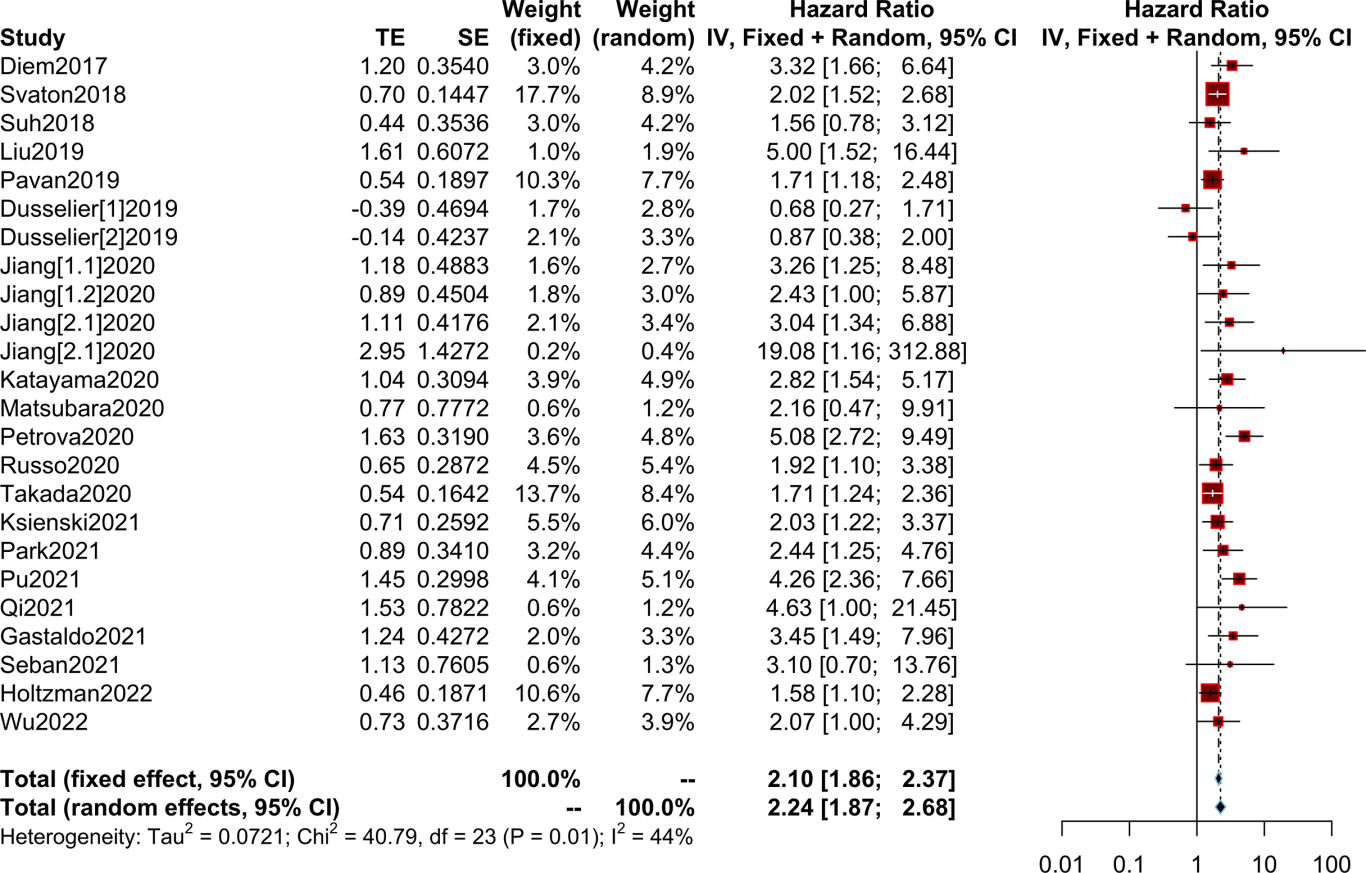
Meta-analysis of the relationship between different comparative models of the platelet to lymphocyte ratio (PLR) and overall survival (OS).

**Figure 4:**
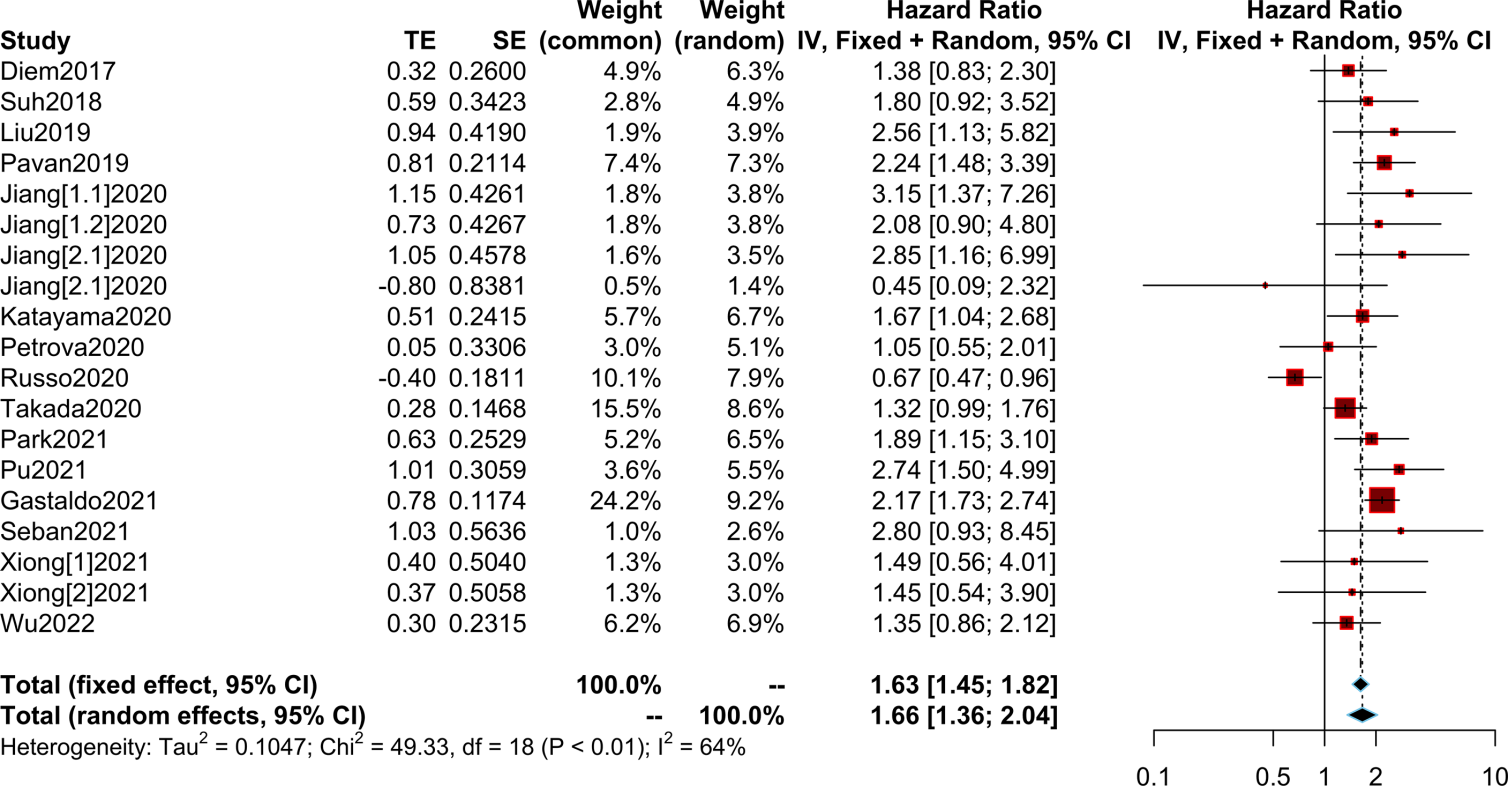
Meta-analysis of the relationship between different comparative models of the platelet to lymphocyte ratio (PLR) and progression-free survival (PFS).

We collected OS/PFS data from each study. The overall median OS was 12 months (range, 8.4-NR months), while the median PFS was 5.25 months (range, 2-8.6 months). The median OS was 8.45 months (range, 2.99-14.7 months) in patients with high PLR and 15.2 months (range 10.6-36.4 months) in patients with low PLR. Furthermore, the median PFS was 3.4 months (range, 1.4-5.2 months) in patients with high PLR, while it was 6.95 months (range, 3-9.16 months) in patients with low PLR (**Supplementary Table S5**). After the synthesis of the HRs for OS from 20 studies, we observed that high PLR was associated with poorer OS (HR=2.24; 95% CI: 1.87-2.68; I² =44%, P=0.01) (**Figure 3**). After combining HRs for PFS from 15 studies, we observed that high PLR was also associated with adverse PFS (HR=1.66; 95% CI: 1.36-2.04; I²=64%, P<0.01) (**Figure 4** **)**.

### Subgroup analyses for OS and PFS

**Figure 5:**
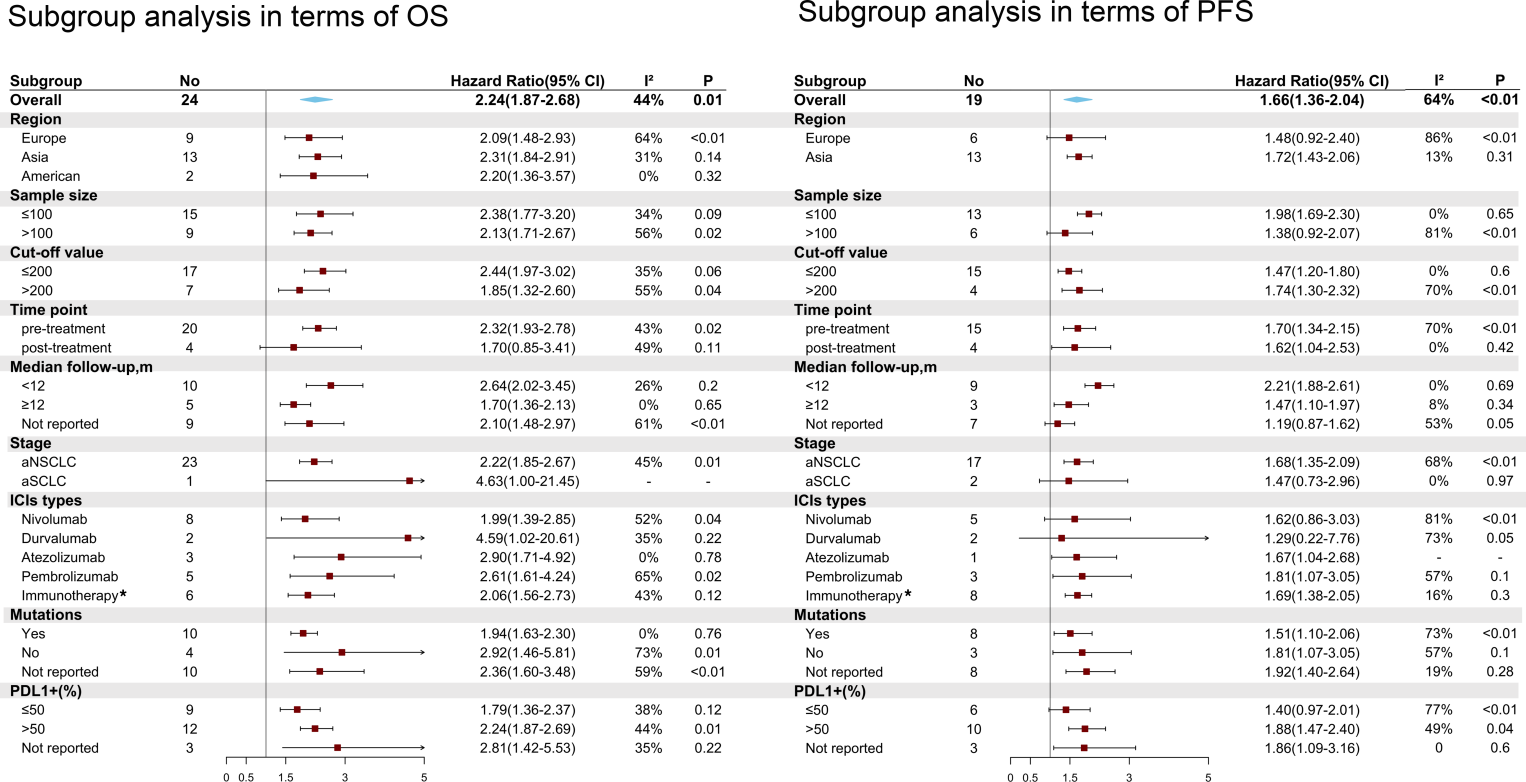
Subgroup analyses of the associations between the platelet to lymphocyte ratio (PLR) and overall survival (OS) and progression-free survival (PFS). *Immunotherapy means these articles reporting different types of ICIs or no detailed types of ICIs.

In subgroup analyses, pretreatment high PLR was significantly associated with poorer OS (HR=2.32; 95% CI: 1.93-2.78; I² =43%; P=0.02) and PFS (HR=1.70; 95% CI: 1.34-2.15; I² =70%; P<0.01). Furthermore, high PLR in terms of different cutoff values correlated with poorer OS and PFS, but a lower PLR (cutoff <200) achieved lower heterogeneity (I² = 35% for OS and I² = 0% for PFS). High PLR was significantly associated with adverse OS (HR=2.22; 95% CI: 1.85-2.67; I² =45%; P=0.01) and PFS (HR=1.68; 95% CI: 1.35-2.09; I² =68%; P<0.01) in advanced NSCLC; in contrast, PLR was not associated with OS (HR=4.63; 95% CI: 1-21.45); or PFS (HR=1.47; 95% CI: 0.73-2.96; I² =0%; P<0.97) in advanced SCLC. In addition, low PLR could benefit OS irrespective of the regions, types of ICIs and proportion of patients with PD-L1+. However, high PLR from the European region was not significantly associated with poorer PFS (HR=1.48; 95% CI: 0.92-2.40, I² =86%; P<0.01), and the same negative results were observed for nivolumab (HR=1.62; 95% CI: 0.86-3.03, I² =81%; P<0.01), durvalumab (HR=1.29; 95% CI: 0.22-7.76, I² =73%; P=0.05) and a small proportion of patients with PD-L1+ (PDL1+1 ≤ 50% group) (HR=1.40; 95% CI: 0.97-2.01, I² =77%; P<0.01). Interestingly, in the group of patients with driver gene mutations, elevated PLR still showed a significant association with adverse OS (HR=1.94; 95% CI: 1.63-2.30; I² =0%; P=0.76) and PFS (HR=1.51; 95% CI: 1.10-2.06; I² =73%; P<0.01) (**Figure 5**).

### Sensitivity analysis and publication bias

**Figure 6:**
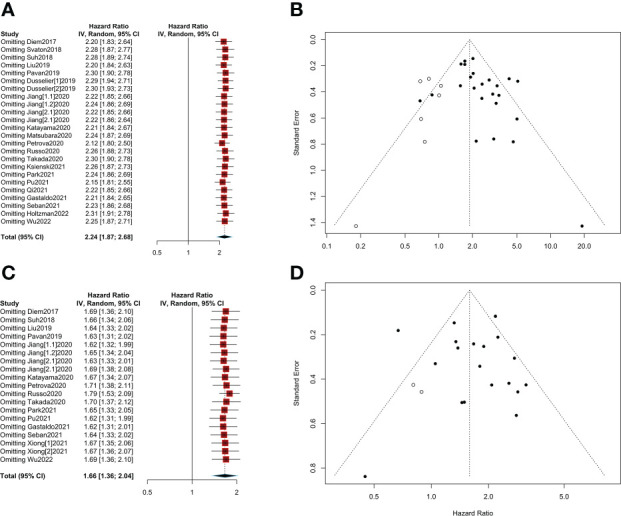
Sensitivity analyses and publication bias. **(A)** Sensitivity analysis of overall survival (OS); **(B)** A trim and fill funnel plot of overall survival (OS); **(C)** Sensitivity analysis of progression-free survival (PFS); **(D)** A trim and fill funnel plot of progression-free survival (PFS).

Sensitivity analysis showed that pooled HRs for OS and PFS were stable after excluding each of the included studies (HR>1, **Figures 6A, C**). No evidence of obvious publication bias was found in Begg’s test or Egger’s test (p>0.05, **Supplementary Figure S1**). Finally, trim and fill funnel plots of OS and PFS showed the same results after eliminating the potential publication bias, and the HR was 1.88 (95% CI: 1.53-2.33) for OS and 1.59 (95% CI: 1.30-1.94) for PFS (**Figures 6B, D**).

## Discussion

Since the role of the platelet-lymphocyte ratio (PLR) in predicting the efficacy of immunotherapy in advanced lung cancers is disputable, this study gathered 21 studies including 2312 patients with advanced lung cancer receiving ICIs to address this concern. Overall, the final meta-analysis showed that patients with high PLR were associated with poor OS and PFS, whereas patients with low PLR showed better OS and PFS. Furthermore, patients with high PLR achieved lower ORR and DCR than those with low PLR. Overall, these evidences showed that PLR was a practical prognostic biomarker for immunotherapy in advanced lung cancer.

Recently, several meta-analyses with respect to the prognostic value of PLR in cancer immunotherapy were published. Xu et al (39) reported that low PLR may be related to better survival for cancer patients with melanoma and NSCLC, which was consistent with our results. Another meta-analysis performed by Tan et al. (40) showed that PLR did not achieve a significant association with clinical outcomes in patients treated with immunotherapy. However, they included only four retrospective articles that evaluated the correlation between PLR and immunotherapy in cancer patients. High heterogeneity from their results was likely to reduce the reliability.

To detect the source of heterogeneity as well as the potential predictive value of PLR in different groups, further subgroup analyses were performed in our meta-analysis. These results suggested that pretreatment high PLR was significantly associated with adverse OS and PFS. A recent meta-analysis performed by Liu et al. (41) also indicated a similar trend to ours, but the results of their subgroup analyses showed no significant correlation between posttreatment PLR and poor OS and PFS, which is inconsistent with our results. Our meta-analysis suggested that posttreatment PLR was significantly associated with PFS and with lower heterogeneity. Although high PLR in terms of different cutoff values correlated with poorer OS and PFS, a lower PLR (cutoff <200) achieved lower heterogeneity. This result indicated that the cutoff value of PLR set within 200 may be more reasonable. A meta-analysis conducted by Xu et al (39) suggested that PLR ≤ 170 was significantly correlated with PFS, which further supported our results.

In addition, high PLR was significantly associated with poor OS and PFS in advanced NSCLC; however, high PLR was not associated with adverse OS and PFS in advanced SCLC. Since only two included articles reported OS or PFS for advanced SCLC, the negative results for advanced SCLC may be biased. More investigations are warranted to provide evidences for the predictive value of PLR in advanced SCLC. Furthermore, PLR could predict poorer OS irrespective of regions, types of ICIs and proportion of patients with PD-L1+. However, high PLR from the European region was not associated with poorer PFS, and the same negative results were observed with nivolumab, durvalumab and a small proportion of patients with PD-L1+. These negative results may be the source of the high heterogeneity of PLR in predicting PFS, and these factors may potentially affect the prognostic value of PLR in PFS. Several factors, such as tumor mutation load and driver gene mutation, may affect the response to immunotherapy (42). Previous articles stated that driven gene mutations, including EGFR+, were associated with a poor response to immunotherapy (43). Our subgroup analysis showed that high PLR was associated with adverse OS/PFS in the presence of driven gene mutations in the cohort, which indicated that PLR was an effective marker for immunotherapy irrespective of driven gene mutations.

In summary, in contrast to the previous meta-analysis, we collected more original articles. Besides, more subgroup analyses were conducted in terms of clinical information to detect sources of heterogeneity and the potential predictive value of PLR in different groups.

In addition to PLR, it was reported that other inflammatory biomarkers (neutrophil to lymphocyte ratio (NLR) and lymphocyte to monocyte ratio (LMR), etc.) were associated with the prognosis of immunotherapy in advanced lung cancer. Elevated NLR was associated with poorer OS and PFS in patients with lung cancer receiving immunotherapy in a previous meta-analysis (9, 44). Furthermore, the meta-analysis from Liu et al (41) showed that a low LMR is related to unsatisfactory survival outcomes in NSCLC patients treated with ICIs. In addition, these inflammatory biomarkers were also reliable in predicting the outcome of other advanced tumors treated with immunotherapy. Elevated NLR was observed to be associated with adverse OS and PFS in patients with metastatic renal cell carcinoma (45) and metastatic melanoma (46) treated with ICIs in a previous meta-analysis. Of note, the predictive value of PLR was also observed in other advanced cancers (melanoma (47), renal cell carcinoma (48)) treated with immunotherapy. However, further investigations are warranted to confirm the prognostic value of PLR in other tumors.

Platelet elevation accelerates tumor progression by promoting the formation of new blood vessels and the production of adhesion molecules (49–51). In the latest research, Hinterleitner et al. (52) found that PD-L1 protein was able to be transferred from NSCLC tumor cells to platelets, and platelets expressing PD-L1 inhibited the infiltration of CD4+ and CD8+ T lymph cells. In contrast, lymphocytes play an important role in the antitumor process by releasing a range of cytokines that activate antitumor immunity (53). In summary, peripheral platelets may reflect the expression of PD-L1 on tumors, and high PLR may impair the efficacy of ICIs. These findings provide some evidence of the predictive value of immunotherapy for PLR. However, it is known that all individuals show a greatly different response to immunotherapy. Although PLR showed a potential predictive value in immunotherapy, this indicator should not be used alone. PLR combined with other biomarkers may improve the efficacy of predictive value, but further studies are needed to confirm the efficacy of predictive value of PLR alone or combined with other biomarkers.

There were some limitations in our study (1): the type of included studies was retrospective, which is not convincing compared with clinical randomized controlled trials (RCTs) (2). Because of the small number of samples, the heterogeneity of the combined results is inevitable. However, we tried to reduce this heterogeneity through various subgroup analyses. Except for the posttreatment value of PLR, the combined HRs for OS maintained good consistency. In contrast, we found that the heterogeneity of PFS may be related to regions, sample size, ICI types, median follow-up time and proportion of patients with PD-L1+. However, PLR still showed a robust correlation with PFS after sensitivity analysis (3). In addition, the cutoff value of PLR varied greatly. Although the cutoff values of PLR did not affect the ultimate result of OS/PFS in our study, the optimal cutoff value should be taken into priority consideration before the application of PLR.

## Conclusion

On the whole, our meta-analysis showed that patients with low PLR had better OS and PFS, as well as higher ORR and DCR when receiving immunotherapy in advanced lung cancer especially for advanced NSCLC. PLR showed a great potential value in predicting the outcome of immunotherapy in advanced NSCLC; further investigations are warranted to confirm the prognostic value of the PLR in advanced SCLC.

## Data availability statement

The original contributions presented in the study are included in the article/**Supplementary Material**. Further inquiries can be directed to the corresponding authors.

## Author contributions

KZ and JDM designed the study. KZ, JC, and HHL designed the statistical plan. KZ, LCL, and ZZS performed the key analyses. LW, ZYP, and KZ generated and collected the data. LW, ZYP, and LCL assisted in data interpretation. KZ wrote the manuscript. JDM and JC revised the manuscript. All authors contributed to the article and approved the submitted version.

## Funding

This work was supported by a grant from the 1.3.5 Project for Disciplines of Excellence (ZYJC18009), West China Hospital, Sichuan University to Dr. Mei.

## Conflict of interest

The authors declare that the research was conducted in the absence of any commercial or financial relationships that could be construed as a potential conflict of interest.

## Publisher’s note

All claims expressed in this article are solely those of the authors and do not necessarily represent those of their affiliated organizations, or those of the publisher, the editors and the reviewers. Any product that may be evaluated in this article, or claim that may be made by its manufacturer, is not guaranteed or endorsed by the publisher.

## Abbreviations

PLR, platelet-to-lymphocyte ratio; NSCLC, non-small cell lung cancer; SCLC, small cell lung cancer; OS, HR, hazard ratio; OR, odds ratio; CI, confidence interval; PFS, progression-free survival; OS, overall survival; ORR, objective response rate; DCR, disease control rate; ICIs, immune checkpoint inhibitors; TMB, tumor mutational burden; PD-L1, programmed cell death ligand 1; TPS, tumor proportion score; NOS, Newcastle–Ottawa Scale; EGFR, epidermal growth factor receptor; ALK, anaplastic lymphoma kinase; NLR, neutrophil-to-lymphocyte ratio; LMR, lymphocyte-to-monocyte ratio. N, nivolumab; P, pembrolizumab; A, atezolizumab; T, toripalimab.
